# St. Louis Medical and Surgical Journal

**Published:** 1850-05

**Authors:** 


### ST. LOUIS MEDICAL AND SURGICAL JOURNAL.

We are gratified in receiving once more this valued journal, and in welcoming into the editorial brotherhood Prof. John B. Johnson, in the place of Prof. McDowell, who has retired.

---

We hope to lay before our readers, in the next number, a synopsis of the proceedings of the American Medical Association, at their meeting in Cincinnati, on the 6th inst.

